# Sleep in honey bees is affected by the herbicide glyphosate

**DOI:** 10.1038/s41598-020-67477-6

**Published:** 2020-06-29

**Authors:** Diego E. Vázquez, M. Sol Balbuena, Fidel Chaves, Jacob Gora, Randolf Menzel, Walter M. Farina

**Affiliations:** 1 Laboratorio de Insectos Sociales, Departamento de Biodiversidad y Biología Experimental, Facultad de Ciencias Exactas y Naturales, Universidad de Buenos Aires, Buenos Aires, Argentina; 2 Instituto de Fisiología, Biología Molecular y Neurociencias (IFIBYNE), CONICET-Universidad de Buenos Aires, Buenos Aires, Argentina; 3 Institut für Biologie, Freie Universität Berlin, Berlin, Germany

**Keywords:** Zoology, Animal behaviour, Animal physiology

## Abstract

Sleep plays an essential role in both neural and energetic homeostasis of animals. Honey bees (*Apis mellifera*) manifest the sleep state as a reduction in muscle tone and antennal movements, which is susceptible to physical or chemical disturbances. This social insect is one of the most important pollinators in agricultural ecosystems, being exposed to a great variety of agrochemicals, which might affect its sleep behaviour. The intake of glyphosate (GLY), the herbicide most widely used worldwide, impairs learning, gustatory responsiveness and navigation in honey bees. In general, these cognitive abilities are linked with the amount and quality of sleep. Furthermore, it has been reported that animals exposed to sleep disturbances show impairments in both metabolism and memory consolidation. Consequently, we assessed the sleep pattern of bees fed with a sugar solution containing GLY (0, 25, 50 and 100 ng) by quantifying their antennal activity during the scotophase. We found that the ingestion of 50 ng of GLY decreased both antennal activity and sleep bout frequency. This sleep deepening after GLY intake could be explained as a consequence of the regenerative function of sleep and the metabolic stress induced by the herbicide.

## Introduction

Sleep is a reversible behavioural state, usually associated with quiescence, in which animals present elevated sensory thresholds that allow them to partially disconnect from the external world^1,2^. To some extent, this state is important for synaptic homeostasis, regeneration and energy conservation^3,4^. Besides, sleep occurs cyclically presenting an endogenous rhythm synchronized with environmental factors^5^. Therefore, neuroendocrine signalling pathways are involved in the regulation of photoperiodism and rhythmicity^5–7^.

In the same way as vertebrates, insects choose resting places and adopt specific stereotypical postures while asleep^1,2,8^. In honey bees (*Apis mellifera*), sleep can be unequivocally deduced from the movement of their antennae and their body posture^9–11^. Moreover, their electrophysiological brain activity correlates well with their resting behaviour^12–14^. They are diurnal insects and rest mainly inside the nest, according to the requirements of the colony^15^. Nevertheless, it is not possible to determine a unique and long sleep bout as those commonly reported for mammals^11,16^. These social insects display numerous sleep bouts (around 50 on average) interrupted by brief stages of awakening where they are immobile or grooming^11^. The maximum duration of a sleep bout of forager bees ranges from 10 to 15 min on average during the nightly rest^11^. In colony and laboratory assays, three sleep stages have been described in honey bees, similarly to those reported for mammals^9–11,16^. Between wakefulness and deep sleep stages, there is a light sleep stage that seems to be a transitory period. It has the shortest bout duration in which bees exhibit spontaneous antennal movements and they are more sensitive to light stimuli. Meanwhile, deep sleep exhibits bouts without antennal movements, with an increase in the duration of the ventilation cycle^10^ and a decrease in body temperature^9^. Besides, the hourly amount of antennal quiescence has a maximum peak in the middle of the rest^10^. However, there are differences in the sleep pattern when bees get older^16^. Young bees are mostly active around-the-clock with no circadian rhythm for sleep, while foragers are active during the day visiting flowers and resting during the night with a strong circadian rhythm^11^.

In honey bees, it has been shown that deprivation or disturbances of sleep impair the encoding of information and memory consolidation^8,17,18^. Particularly, they affect the precision of the waggle dance^19^ and navigation abilities^8^. The nature of those sleep stressors is diverse, they can be related to the internal state of the individual (e.g., starvation) or its surrounding environment (e.g., vibrations, light, temperature, ecological interactions)^20^. Moreover, chemical agents can also disturb the resting–awakening cycles of insects. In this sense, caffeine promotes wakefulness in fruit flies^21^, whereas anaesthetics promote resting behaviour in honey bees^22^.

Since the honey bee *Apis mellifera* is one of the most important pollinators in agricultural ecosystems, it is exposed to a great variety of agrochemicals^23^. The knowledge about the impact of these substances in the animal resting behaviour is sparse. Only one previous study reported induction of sleep apnea in rats exposed to the insecticide chlorpyrifos^24^. One of the widest agrochemicals used worldwide is the herbicide glyphosate (GLY)^25^. GLY is considered as a low toxicity pesticide for honey bees, however, research has shown that it affects their behaviour and physiology^26,27^. As we mentioned, sleep plays an essential role in honey bee memory consolidation^8,18,28^ and there is evidence that GLY in chronic exposures impairs the associative learning and cognitive abilities of these pollinators^26^. Therefore, we set out to evaluate the effect of the intake of non-lethal amounts of GLY on the sleep of forager honey bees. For that, the antennal activity during the nightly rest was recorded under controlled laboratory conditions.

## Results

**Figure 1:**
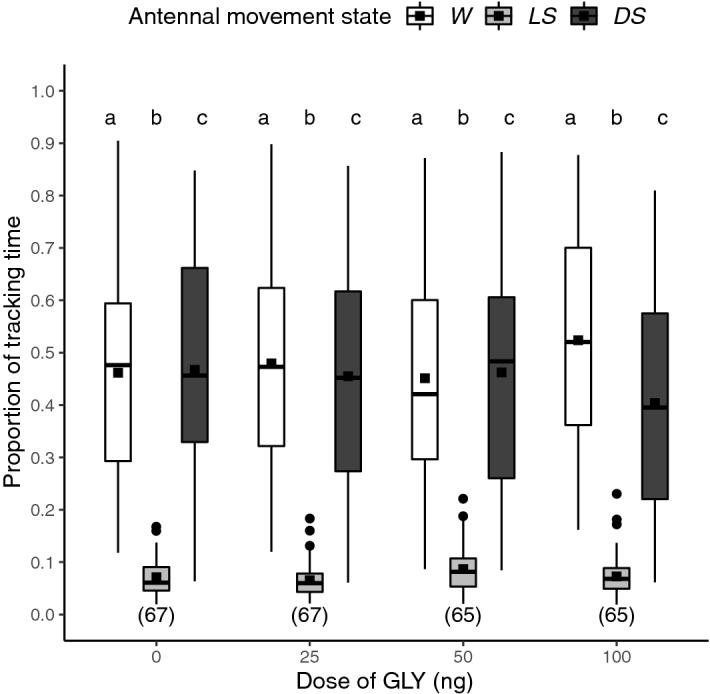
Glyphosate does not affect the proportion of time invested in rest. Dispersion of proportions of time invested per forager bee in each stage of the resting–awakening cycle (wakefulness/W: white, light sleep/LS: grey, and deep sleep/DS: black) during scotophase (18:00–6:00) according to GLY exposure. Acute exposure to contaminated food with the following doses of GLY per group: 0, 25, 50 and 100 ng. The number of assessed bees per group is shown in brackets. Different letters indicate significant differences among cycle stages (P < 0.05) (GLMM model: prop. of time ~ [GLY] + cycle stage + (1|day/bee), Tukey test in Supplementary Table S3).

**Figure 2:**
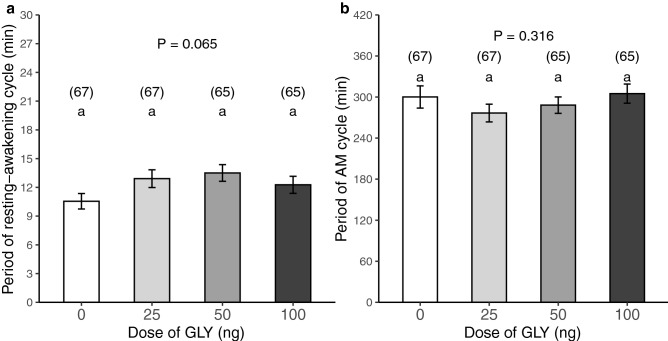
Glyphosate slightly affects sleep frequency. (**a**) Average dominant duration of each stage (wakefulness or sleep) in the resting–awakening cycle displayed in forager bees during scotophase (18:00–6:00) according to GLY exposure (mean ± SEM). (**b**) Average dominant duration of the amplitude modulation (AM) in the resting–awakening cycle. Acute exposure to contaminated food with the following doses of GLY per group: 0, 25, 50 and 100 ng. The bars are plotted with a greyscale gradient for increasing doses of GLY. The number of assessed bees per group is shown in brackets. Groups with different letters have significantly different means (P < 0.05) (GLMM model: dominant period ~ [GLY] + (1|day), Tukey test in SI Table S4).

We assessed changes in the sleep pattern of forager bees during the scotophase (12 h) after an oral acute exposure to GLY (0, 25, 50 or 100 ng) in the prior photophase. Only the time series with strong signals from the recordings were analyzed (see “Materials and methods”). To assess a global sleep pattern, we calculated the proportion of time invested in each cycle stage during the overall recording time (Fig. 1). As a result, there were significant differences among the three cycle stages for all treatments but GLY exposure did not induce significant differences in the global sleep pattern (GLMM model: prop. of time ~ [GLY] + cycle stage + (1|day/bee). Variance structure: < 0.01% among bees and within days.[GLY] term: χ^2^ (6,7) = 0.04, P = 0.998. Cycle stage term: χ^2^ (2,4) = 632.58, P < 0.001, N = 264, for *post-hoc* pairwise comparisons see SI Table S1). During scotophase, bees slept around half of the time and had a fast transition between wakefulness and quiescence (median proportion of time for each stage in the control group: 48% in wakefulness, 6% in light sleep and 46% in deep sleep). Furthermore, the oscillating bouts of awakening–resting (R–A) occurred in short dominant periods with an average duration of 10.55 ± 6.61 min in the testing signal of the control group (Fig. 2a). However, there were no significant differences among treatments (GLMM model: dominant period of R–A cycle ~ [GLY] + (1|day). Variance structure: < 0.01% among days. [GLY] term: F (3, 6) = 7.22, P = 0.065, N = 264, for post-hoc pairwise comparisons see SI Table S4). Notwithstanding, it is important to stress that honey bees exposed to food containing 50 ng of GLY showed a marginal significance to large dominant periods (or small dominant frequency) with average durations of 13.5 ± 7.01 min.

Another aspect worth considering was the signal modulation. In this sense, testing signals obtained from the honey bees showed modulation in the amplitude (AM) of the antennal activity (changes in the magnitude of the activity during sequential wakefulness periods) but not in its frequency (FM). The pattern was detected when we analyzed the shape of each testing signal in both periodogram and spectrogram. This modulation process also oscillated and had a dominant period with an average duration of 300 ± 133.2 min in the envelope of the testing signal of the control group. Nevertheless, there were no significant differences in that modulation signal due to the intake of food with GLY (Fig. 2b. GLMM model: dominant period of AM cycle ~ [GLY] + (1|day). Variance structure: < 0.01% among days. [GLY] term: F (3, 6) = 3.54, P = 0.316, N = 264).

**Figure 3:**
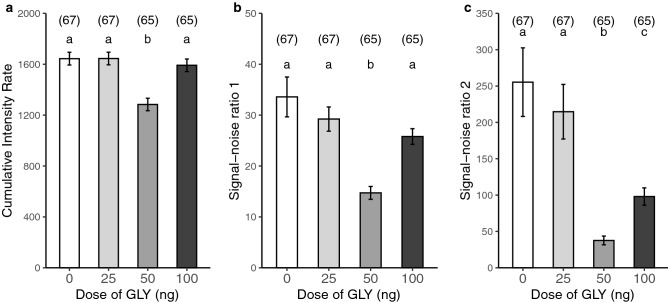
Glyphosate affects antennal activity during the wakefulness. (**a**) Average cumulative intensity rate (total antennal activity/lifetime) during the resting–awakening cycle (predominantly wakefulness stage) displayed during scotophase (18:00–6:00) according to GLY exposure (mean ± SEM). (**b**) Average signal–noise ratio 1 (Eq. 1) and (**c**) average signal–noise ratio 2 (Eq. 2) calculated for the biological signals recorded in the forager bees during the experiment (mean ± SEM). Acute exposure to contaminated food with the following doses of GLY per group: 0, 25, 50 and 100 ng. The bars are plotted with a greyscale gradient for increasing doses of GLY. The number of assessed bees per group is shown in brackets. Groups with different letters have significantly different means (P < 0.05) (GLMM model: CIR or SNR ~ [GLY] + (1|day), Tukey test in SI Tables S5, S6 and S7).

Lastly, we estimated the intensity of the antennal activity in the wakefulness stage during the scotophase in three different ways. First, the cumulative intensity rate showed a significant reduction of the antennal activity for the group of bees exposed to 50 ng of GLY (Fig. 3a). GLMM model: cumulative intensity rate ~ [GLY] + (1|day). Variance structure: 9.9% among days. [GLY] term: LR(3, 6) = 38.31, P < 0.001, N = 264, for post-hoc pairwise comparisons see SI Table S5). Meanwhile, in the same way, both signal–noise ratios showed a significant reduction of the strength in the biological signal for the groups of bees exposed to 50 and 100 ng of GLY (Fig. 3b,c. GLMM model: SNR1 or SNR2 ~ [GLY] + (1|day). Variance structure of both: < 0.01% among days. For SNR1: [GLY] term: F(3, 6) = 49.90, P < 0.001, N = 264, for *post-hoc* pairwise comparisons see SI Table S6. For SNR2: [GLY] term: F(3, 6) = 95.05, P < 0.001, N = 264, for post-hoc pairwise comparisons see SI Table S7).

To conclude, we noted that the antennal movement of bees which died during the recording was similar on the cumulative intensity rate (see SI Fig. S5; SI Table S6) but significantly different in the proportion of time per cycle stage (less time in deep sleep, see SI Fig. S6; SI Table S7). Besides, 31.3% of control bees died in the mean recording time of 8.05 ± 6.89 h. Nevertheless, our results showed no significant effects on survival and tracking time when we compared among bees exposed to different doses of the herbicide (see SI Fig. S7). Therefore, the doses administered to the forager bees were sub-lethal (CPH model: survival ~ [GLY] + strata(day), χ^2^ (3) = 2.61, P = 0.455, N = 264).

## Discussion

The acute intake of GLY in sub-lethal doses (50–100 ng) affected the sleep pattern of forager honey bees, as estimated from their antennal movements under laboratory conditions. Treated foragers showed a marginal significance to spend more time sleeping in each bout of the resting–awakening cycle than untreated bees; however, they sleep the same total time during the scotophase. Consequently, exposed bees have a trend to interrupt less frequently their rest. Moreover, these bees showed antennal hypoactivity in the nightly awakening bouts. Therefore, our findings suggest that honey bees intensify their sleep at night after they intake food contaminated with GLY.

A key feature of animal sleep is its homeostatic regulation which it is partly independent of the circadian clock^1,2,29^. Substances such as caffeine or antihistamines alter sleep intensity or latency but do not affect sleep timing^2^. Apart from that, a long period of awake or diurnal experiences of intense learning and neural plasticity can induce sleep pressure^29^. Honey bees exhibit prolonged sleep after navigation learning, supporting the role of sleep in spatial memory consolidation^8^. Stressors can affect sleep deepness, often in the absence of changes in sleep duration. Recovery sleep after deprivation in fruit flies displays a higher arousal threshold than during baseline sleep, and it is less fragmented with a decrease in the number of brief awakenings^30^. Also, honey bees and rats compensate sleep deficits or stressful diurnal activity by sleep deepening in the following scotophase^8,17,31,32^. During this deepening, the sleep-disturbed bees display a decrease of the antennal activity accompanied by an increase of the duration of sleep bouts as seen in our experiment. Few studies have faced the homeostatic restorative value of quiet wakefulness and sleep deepening. On one hand, it has been proposed that sleep may allow the removal of toxic free radicals accumulated in the brain during wakefulness^33,34^. On the other hand, in human and rats sleep recovery acts as a regenerative function in the muscle when stress hormones and metabolites are restored to basal levels^35,36^.

The machinery for sleep homeostasis is modulated by the complex interaction of neuronal circuits and neuroendocrine signalling pathways^5–7^. Changes occur in brain gene expression during the resting–awakening cycle. Wakefulness stage leads to up-regulation of transcripts involved in mobilizing energy stores, in response to cellular stress and in facilitating synaptic potentiation^2,4^. Conversely, during sleep different transcripts are up-regulated: the ones involved in protein synthesis, lipid metabolism, and synaptic consolidation or downscaling^2,4^. These homeostatic regulations are in line with the energy conservation and regenerative function of sleep. Thus, the resting state is physiologically regulated by neuromodulators that interact with the metabolism of the animal and with a variety of stimuli from the environment. The strongest wake-promoting neuromodulators in mammals and insects are catecholamines (e.g. dopamine, norepinephrine and octopamine)^2,5^. Meanwhile, some examples of sleep-promoting neuromodulators include serotonin, SIFamide, sNPF and allatostatin A^2,5^. The fact that SIFamide and allatostatin A modulate both feeding behaviour and resting state indicates a strong link between metabolism and sleep^2^. More evidence of this relation is that sleep deprivation increases the metabolic rate in the Pacific beetle cockroach^37^. In the same vein, octopamine shows both functions to control insect sleep and metabolism by interacting with the insulin pathway^38^. The hemolymph level of this neurohormone is modulated by metabolic stress affecting different behaviours in honey bees such as hive maintenance, foraging and sensory input^7^. Indeed, changes in the sleep pattern correlate with metabolic stress in fruit flies. During sleep deprivation its metabolic rate increases while in sleep rebound or pharmacologically induced sleep it decreases^39^.

Studies in both vertebrates and invertebrates proved that GLY induces signs of metabolic stress^27,40–42^. In honey bees, different studies have suggested that chronic exposures to this herbicide can trigger oxidative stress and detoxifying pathways in adults as well as in brood^27,43–46^. Oxidative stress is associated with increased production of free radicals during catabolism and can trigger apoptosis and energy depletion^46,47^. These toxicity signs could be a consequence of the biocide action of the herbicide in the gut microbiota of honey bees^48^. All the energetic demands to accomplish with optimal basal physiology are homeostatically regulated by compensatory mechanisms that can include regenerative processes during sleep. Therefore, the sleep deepening induced by the acute intake of the herbicide could be explained because of the homeostatic function of sleep and metabolic stress. Although these findings do not seem to explain the adverse effects of GLY in the learning and cognitive abilities of honey bees per se^26^, they have a meaningful contribution to the knowledge about the herbicide effects. More studies of the ecological impact of different agrochemicals in the sleep behaviour of pollinators and other agrobionts are necessary.

## Materials and methods

### Study site and animals

The experiment was performed in February–March 2017, during the summer season. Forager bees were captured at an artificial feeder offering 30% w/w sucrose solution. This feeder and the hives of *Apis mellifera* L. were located in the experimental apiary of the University of Buenos Aires, Argentina (34° 32′ S, 58° 26′ W). For 17 days, 24 bees were daily caught in plastic tubes at the same time every day (15:00). Then, they were anaesthetized at − 20 °C during 2 min and harnessed in small metal tubes that restricted body movement but allowed free movement of their antennae and mouthparts, this procedure took half an hour.

### Acute exposure to GLY

To evaluate the effects of GLY on the resting–awakening (R–A) cycle of forager bees, they were exposed to acute doses of the herbicide. Harnessed bees were randomly sorted in four treatments: control (fed with 30% w/w sucrose solution without herbicide) and three groups fed with the same concentration of sucrose solution but with the addition of 1.25, 2.5 or 5 mg a.e. of GLY per litre (analytical standard provided by Sigma-Aldrich, purity of 99.2%). To prepare the food mixture for each concentration, we diluted a stock solution (bidestilled water as solvent) of 100 mg a.e. GLY L^−1^ in sucrose solution. Food of each treatment (20 µL) was administered individually using an automatic multimicropipette (Multipette M4, Eppendorf) and it took less than 10 min from the first bee to the last one. Consequently, the GLY doses for each bee per treatment were: 0, 25, 50, and 100 ng of the herbicide. After feeding, bees were left 1 h inside an incubator (27 °C and 60% RH in darkness. This time allows for the absorption of GLY in the digestive tract and diminishes the stress of immobilization. We assumed that the GLY had time to act during that time independently of the feeding order. The GLY concentrations were chosen according to the highest measurements reported in previous studies from agricultural landscapes and the median expected environmental concentration (reviewed by Farina et al.^26^), assuming the worst-case exposure scenario.

### Recording setup and data acquisition

The antennal movement of the harnessed bees was analyzed since it is a good indicator of the R–A cycle in honey bees^9,10^. Three distinct cycle stages were defined based on previous descriptions^9–11,16^: wakefulness stage (high antennal activity), light sleep stage (low antennal activity) and deep sleep stage (quiescence). The antennal movement was recorded with a device made ad hoc developed by Zwaka et al.^28^. For that, four bees per treatment were chosen randomly from the original 24 experimental subjects to be recorded per day. The extra ones were taken because some of them could die during manipulation. In the device, bees were placed individually within a ventilated chamber (10 × 3.3 × 4 cm) of a plexiglass box and were video-recorded throughout 12 h in darkness with infrared light and steady room conditions. To reflect the actual R–A cycle throughout the experimental time, the recordings started at dusk (18:00) and stopped at early morning (06:00), the period in which honey bee activity normally decreases according to natural night (i.e., scotophase). The device uses a video camera (Logitech HD Pro Webcam C920, Switzerland. Objective: CCTV LENS, 2.8–12 mm F1.4, 2 megapixel IR, Japan) paired to a computer with a software ad hoc^28^ that records automatically the movements of 16 bees simultaneously with a sampling frequency of 36.41 frames s^−1^. For this purpose, software applies a motion mask during the recording to estimate single-bee activity. The algorithm of the motion mask distinguishes the pixels of the image that change over time. In a first step, an adaptive Gaussian mixture model for background subtraction was applied to separate the foreground (moving objects) from the background^49,50^. The foreground detection allowed us to track down moving objects (a group of pixels) and focus the image processing. In a second step, the difference between foregrounds in two consecutive frames was calculated to quantify the magnitude of change and was defined as the intensity of the output raw signal. If the frames were equal, there was no motion and zero intensity (see SI Fig. S1). It is worth noting that the plexiglass box is a stimuli-free environment (a dark room without odorants). Consequently, the movement of the mouthparts of bees was not observed in the context of our experiment because mandible movements and proboscis extension are very rare without external stimulation. Therefore, we can conclude that the biological signal was composed only by the antennal movements.

### Processing and analysis of the signal

The output raw signal was corrected to remove non-biological signal using a blank film (without bees) (see SI Fig. S2). In the blank recording, 99.75% of the signal was under 10 intensity units. Consequently, the registered antennal activity under this threshold was corrected as zero (henceforth, testing signal) where bees with motion equal to background baseline were accounted as quiescent. We defined the threshold for light sleep for all bees as the average of the low activity limit of the control group (i.e., an average of the 50% of the mean intensity per bee). Mortality was also recorded during the experiment: bees were considered dead if they were quiescent more than 15 min, and it was also confirmed the morning after. The cumulative intensity of antennal movement (the sum of all values in the testing signal) was relativized to the lifetime during video recording (cumulative intensity rate).

### Periodicity of the signal

Data analysis and graphics were performed in R software (see Supplementary Methods). The periodicity of each testing signal was confirmed with a correlogram using a confidence interval of 95% (see SI Fig. S3). Then, to evaluate the R–A cycle of the experimental bees, we filtered the testing signal to attenuate other periodic processes (i.e., denoising). For that, we centred the signal to zero and applied a filter function (Butterworth family order 1) with a passband between periods from 1 to 30 min. This eliminates undesirable components of the signal from analyses with very short periods such as noise or other biological processes, and very large periods as trends of the main signal or infrequent mouthparts movements. Each filtered signal (mainly oscillation between awakening and resting bouts) was analyzed with spectral analysis (i.e., applying a Fourier Transformation) that allowed us to estimate the dominant period/frequency and the associated power in the signal. The output of the transformation was represented graphically in a periodogram (see Supplementary Methods and SI Fig. S1). It must be taken into account that the two stages of sleep (light and deep) are coupled in the same resting bout for the spectral analysis. This analysis assumes a periodic and stationary signal as input (i.e., a constant dominant period over time). By making use of the spectrogram we evaluated that assumption and signs of modulation in frequency (FM) or amplitude (AM) in the periodic signal (see Supplementary Methods and SI Fig. S1).

### Modulation of periodicity

To analyze differences in amplitude modulation^10^ among treatments, we calculated the envelope of each testing signal (i.e., a smooth curve outlining the extremes of the oscillating signal). Then, we filtered the envelope (Butterworth family order 1) with a passband between periods from 30 min to 6 h (half the length of the sampling time). This denoising signal kept the modulation wave that has a period longer than the carrier wave (in that case the R–A cycle). Each filtered envelope was processed with spectral analysis estimating the dominant period (see SI Fig. S4).

### Data validation

All the analyses were carried out in registers of motion with a relevant biological signal. Hence, we only evaluated bees with strong signal comparing to the blank film (see SI Fig. S2) and above the limit of detection (LOD). For this purpose, we calculated two signal–noise ratios (SNR). The SNR1 calculated from the testing signal (Eq. 1) and the SNR2 calculated from the spectral analysis (Eq. 2). The register of motion was validated when both SNR1 was higher than three (LOD) and SNR2 was higher than two (strength).

1 $$SNR1=\frac{mean\, intensity\, of\, testing\, signal}{standard\, deviation\, of\, blank\, signal}$$

2 $$SNR2= \frac{cumulative\, power\, of\, testing\, signal}{cumulative\, power\, of\, blank\, signal}$$

### Statistics

Data analyses and graphics were performed in R software (see Supplementary Methods). Survival data were analyzed with cox proportional hazard model (CPH). A descriptive analysis was carried out in each temporal series of data to summarize its periodic behaviour (see above). The summary variables were analyzed with generalized linear mixed models (GLMM) using Beta, Gamma or Gaussian distributions for error structure when appropriate. The alpha level was set at 0.05 and p value was corrected with Bonferroni procedure for multiple post hoc pairwise comparisons (Tukey test).

## Supplementary information

Supplementary information
