# Supplementary material for: Sleep in honey bees is affected by the herbicide glyphosate
Source: Sci Rep. 2020 Jun 29;10:10516. doi: 10.1038/s41598-020-67477-6 (PMC7324403; doi:10.1038/s41598-020-67477-6)
Supplement: Supplementary file 1 — Supplementary information [file 41598_2020_67477_MOESM1_ESM.docx]

Supplementary Information for

**Sleep in honey bees is affected by the herbicide glyphosate**

Diego E. Vázquez^a,b,1^, M. Sol Balbuena^a,b,1^, Fidel Chaves^a,b,1^, Jacob Gora^c^, Randolf Menzel^c^ and Walter M. Farina^a,b,*^

^a^ Universidad de Buenos Aires, Facultad de Ciencias Exactas y Naturales, Departamento de Biodiversidad y Biología Experimental, Laboratorio de Insectos Sociales, Buenos Aires, Argentina.

^b^ CONICET-Universidad de Buenos Aires, Instituto de Fisiología, Biología Molecular y Neurociencias (IFIBYNE), Buenos Aires, Argentina.

^c^ Institut für Biologie, Freie Universität Berlin, Berlin, Germany.

^1^ These authors have contributed equally to this work.

^*^ Walter M. Farina

**Email**: [walter@fbmc.fcen.uba.ar](mailto:walter@fbmc.fcen.uba.ar)

**Keywords**

*Apis mellifera*, agrochemical, sleep, antennal movement, energetic homeostasis

**This PDF file includes:**

Supplementary Methods

Supplementary Figures S1 to S7

Supplementary Tables S1 to S7

SI References

**Supplementary Methods**

**An extended technical description of the mathematical models.**

**Periodicity of the signal.**

Data analysis and graphics were performed in R version 3.6.1 [1, 2]. We recorded the antennal movement of harnessed bees as an indicator of the resting-awakening cycle. The setup used a video camera paired to a computer with a software *ad ho*c [3] that recorded automatically the intensity of the antennal movement during 12 h (*T*) with a sampling frequency (*sf*) of 36.41 Hz (or frames/s) (Fig. S1a). Since bees move their antennae several times in a second, this *sf* allows us to avoid aliasing phenomenon [4, 5]. In our experiment, signals with cycle periods smaller than 0.06 s suffered from aliasing. This phenomenon predicted by the Nyquist-Shannon sampling theorem describes the effect of undersampling of a continuous signal (with a frequency higher than half of the *sf*) which can generate an artefact in the signal with false frequencies [4, 5].

After data acquisition, the output raw signal (Fig. S1b) was corrected (henceforth, testing signal) to remove the non-biological component of the signal using a blank recording (without bees, Fig. S2). Testing signals were time serial data with around 1.6 million observations per honey bee. The extraction of descriptive parameters of the time series with reliable information on periodicity and robustness of the biological signal is central to its interpretation. First, the significant periodicity of each testing signal was confirmed by an autocorrelation analysis (Fig. S3) [6, 7, 8]. When there are repeated measures on an experimental unit (each honey bee), it would expect that the observation at time *t* + 1 (a lag) would be quite strongly correlated with the observation at time *t*. For that, we analyzed each time series with the function acf [8]. The autocorrelation and partial autocorrelation coefficients are based on the sample autocovariance (lag 0 is fixed at 1 by convention). The partial correlation coefficients were estimated by fitting autoregressive models of successively higher orders up to the maximum lag. If one or some autocorrelation coefficients were significantly different to zero, it meant that there was a serial correlation in the data. This was assessed using a confidence interval of 95% with upper and lower limits given by Z_1-α/2_ / √*n*, where Z is the critical value for the significance level α in the two-tailed standard score test and n is the number of observations (*n* > 30). Besides, there was periodicity if these significant autocorrelation coefficients displayed an oscillating or a decreasing exponential pattern with a trend to zero. If there were no significant coefficients, the time series had serial independence and therefore randomness (white noise) (Fig. S2b) [9].

The length of time required for one full cycle is called a period. Thus, it is the reciprocal of the frequency (*f =* 1/period, in Hz). Each periodic signal was characterized with the spectral analysis, which was based on the decomposition of the time series into a linear combination of sine and cosine functions for a range of frequencies (Fourier transformation) [8, 10, 11]. It allowed determining one frequency that appears particularly strong or dominant. This is equivalent to a finite Fourier series *y(t)* for a discrete-time series described by linear multiple regression with a succession of trigonometric functions as predictor variables. The maximum frequency for the decomposition according to the Nysquit Theorem is half of the *sf* [4, 5]. Meanwhile, Rayleigh frequency is the minimum frequency that can be resolved by a finite time window [12]. Given a record time that is *Τ* seconds long, the minimum frequency that can be resolved is 1/*Τ*. The regression is fitted with *k* predictor variables where *k/n* (*k* cycles per record length) is the frequency:

$$y\left( t \right)=a_{0}+ \sum_{k=1}^{k=\frac{n}{2}-1} [a_{k} cos(\frac{2\pi k t}{n}) + b_{k} sin(\frac{2\pi k t}{n})]+ a_{n/2} cos(\pi t)$$

The cosine parameters (a_k_) and sine parameters (b_k_) are the regression coefficients that represent the degree of correlation between the respective functions and data. The intercept term (a_0_) is the mean of the time series. The contributions of the *k* frequencies to the regression can be estimated as its power: P_k_ = a_k_^2^ + b_k_^2^. Before this transformation, we filtered the testing signal (black line in Fig. S1c) to attenuate other periodic processes of no interest (i.e., denoising) [13]. For that, we centred the signal to zero (a_0_ = 0) and applied a filter function (Butterworth family for the transfer function with order n = 1) with a passband between cycle periods from 1 to 30 minutes. For smaller values of n, the cutoff will be less sharp [14]. The denoising was carried out using the bwfilter function of the seewave package [15].

Each filtered testing signal was analyzed applying a Fast Fourier Transformation (FFT) that allowed us to estimate the dominant period and its associated power. The output of the transformation was represented graphically in a periodogram which quantifies the contributions of the individual frequencies to the time series regression (Fig. S1d). These values (power) can be interpreted in terms of variance of the data at the respective frequency. If there is a unique large power value, we can conclude that there is a strong periodicity associated with the respective frequency (or period) in the data. Consequently, the white noise spectrum is a horizontal line because of variance is not preferentially concentrated in any particular frequency range (Fig. S2c). The detrending, smoothing and FFT of the time series was performed and plotted using the function spectrum [8]. We subtracted the mean from the time series and its trend; otherwise, the periodogram will mostly be overwhelmed by a very large value for the first cosine coefficient (a_0_). The periodogram distributes the variance over frequency (or period), but it has two drawbacks. The first is that the precise set of frequencies is arbitrary, in as much as it depends on the record length. The second is that the periodogram does not become smoother as the length of the time series increases but just includes more spikes packed closer together. The remedy is to smooth the periodogram, and one way to do this is by using a smoothing kernel of spikes before joining the tips. The argument span in the function is the number of spikes in the kernel. However, the smoothing reduces the heights of peaks, and excessive smoothing blurs the features we are looking for. For this reason, we assessed spectra with different amounts of smoothing and selected the optimum (span = 10). By default, the plot for frequency axis is cycles per sampling interval. It is more intuitive to convert the frequency axis to cycles per unit time and then calculate the period. We should also multiply the spectral density (power values) by 2 so that the area under the periodogram equals the variance of the time series.

These analyses (autocorrelation and spectral) assumes that the time series is a stationary signal as input (i.e., a constant dominant period over time). We used the spectrogram to assess this assumption and signs of modulation in frequency or amplitude [16]. The spectrogram is a plot of a signal in which the vertical axis is frequency, the horizontal axis is tracking time and the amplitude (√power) axis is compressed into a contour map drawn in a colour scale (Fig. S1e). Conventionally, we used red to the strongest signal component, while purple was used to the least strong. The spectrogram was performed using the function spectro of the seewave package [15]. This function corresponds to a short-term Fourier transformation (STFT) with a Hanning window function of the filtered testing signal [17]. The FFT was applied in windows with a length of 100,000 observations with 25% of overlap between two successive windows. The STFTs were normalized (i.e., scaled) by its maximum value. Following the Heisenberg–Gabor limit based on the uncertainty principle, the STFT cannot be precise in both time and frequency [18]. The temporal and frequency precisions of the function are dependent on the window length value. This problem can be reduced in some way with zero-padding (zp = 10) that adds 0 values on both sides of the analysis window. This increases frequency resolution without altering time resolution. The filtered testing signals without modulation should display a continuous horizontal red line at a constant frequency value over time. If there is frequency modulation (FM), the dominant frequency (red line) displays a non-horizontal pattern, e.g. sinusoidal. If there is amplitude modulation (AM), the dominant frequency (red line) displays a non-continuous horizontal pattern like a beat. A beat is an interference pattern between two waves (carrier and modulating) of slightly different frequencies. This pattern is detected as a periodic variation in amplitude whose rate is the difference of the two frequencies.

To analyze the AM process, we calculated the envelope (red line in Fig. S1c) of each filtered testing signal (i.e. a smooth curve outlining the extremes of the oscillating carrier signal) [19]. The Hilbert amplitude envelope was calculated and plotted using the function env of the seewave package [15]. Then, we filtered the envelope (Butterworth family for the transfer function of order n = 1) with a passband between periods from 30 min to 6 h (half the length of the sampling time). Each filtered envelope was processed with spectral analysis estimating the dominant period of modulation as we explain above (Fig. S4).

**Statistics.**

In all response variables obtained from the signal analysis, we evaluated the effect of different doses of GLY as the main fixed factor [8, 20]. However, in the proportion of time and cumulative intensity rate, we additionally tested the cycle stage and survival status as fixed factors.

Survival was analyzed with the Cox Proportional Hazard model (CPH), using the survival package, because we have censoring and stratification (days as strata) [21, 22, 23]. The assumption of a proportional hazard was assessed using the function cox.zph of the same package [24]. The survival curves and confidence intervals were estimated and plotted with the function autoplot of the ggfortify package [25, 26].

With the remaining response variables, we performed statistical analysis with generalized linear mixed models (GLMM) with days as a random factor to take in account the variability among the groups of bees caught in different days [27, 28]. Additionally, in the case of the variable proportion of time, we used the bee factor as nested random factor to meet the independency assumption because we measured the time in each cycle stage in the same individual [27, 28]. The proportion of time was a no frequency-based ratio delimited to a range of values within 0-1. Consequently, we fitted this data to a model with Beta distribution for error structure using the function glmmTMB of the glmmTMB package [29]. Data from continuous variables (dominant period of testing signal and AM signal) were fitted to a model with Gamma distribution for error structure using the function glmer of the lme4 package [30]. These packages fit GLMMs via maximum likelihood. The expression for the likelihood of a mixed-effects model is an integral over the random effects space. For a mixed-effects model with Gaussian distribution, this integral can be evaluated exactly. For the rest of distributions in GLMMs the integral must be approximated and the lme4 package uses the adaptive Gauss-Hermite quadrature. The nAGQ argument controls the number of nodes in the quadrature formula. We set this argument to zero, applying a faster but less exact form of parameter estimation for GLMMs by optimizing the random effects and the fixed-effects coefficients in the penalized iteratively reweighted least-squares step. The data from the signal-noise ratios were analyzed similarly to the continuous variables because they were no frequency-based ratios but not delimited to a range of values within 0-1. Finally, the data from the cumulative intensity rate were fitted to a model with Gaussian distribution for error structure using the function lme of the nlme package [27].

For *post hoc* pairwise comparisons, all level combinations of a significant fixed factor were analyzed with Tukey test using the function glht of the multcomp package [31]. Besides, the selection of the most parsimonious model for CPH and GLMM was carried out assessing the relative importance of each factor with a stepwise subtraction method (approach with hypothesis testing based on comparing nested models) [8, 28] using the functions anova or lrtest, the latter from the lmtest package for the beta regression [32].

For all tests, we report the statistic with its degrees of freedom and p-value when the minimal adequate model was contrasted with the null model. The alpha level was set at 0.05 and p-value corrected for multiple *post hoc* comparisons with Bonferroni procedure (p-value’ = p-value*k, k = number of comparisons) [33, 34].


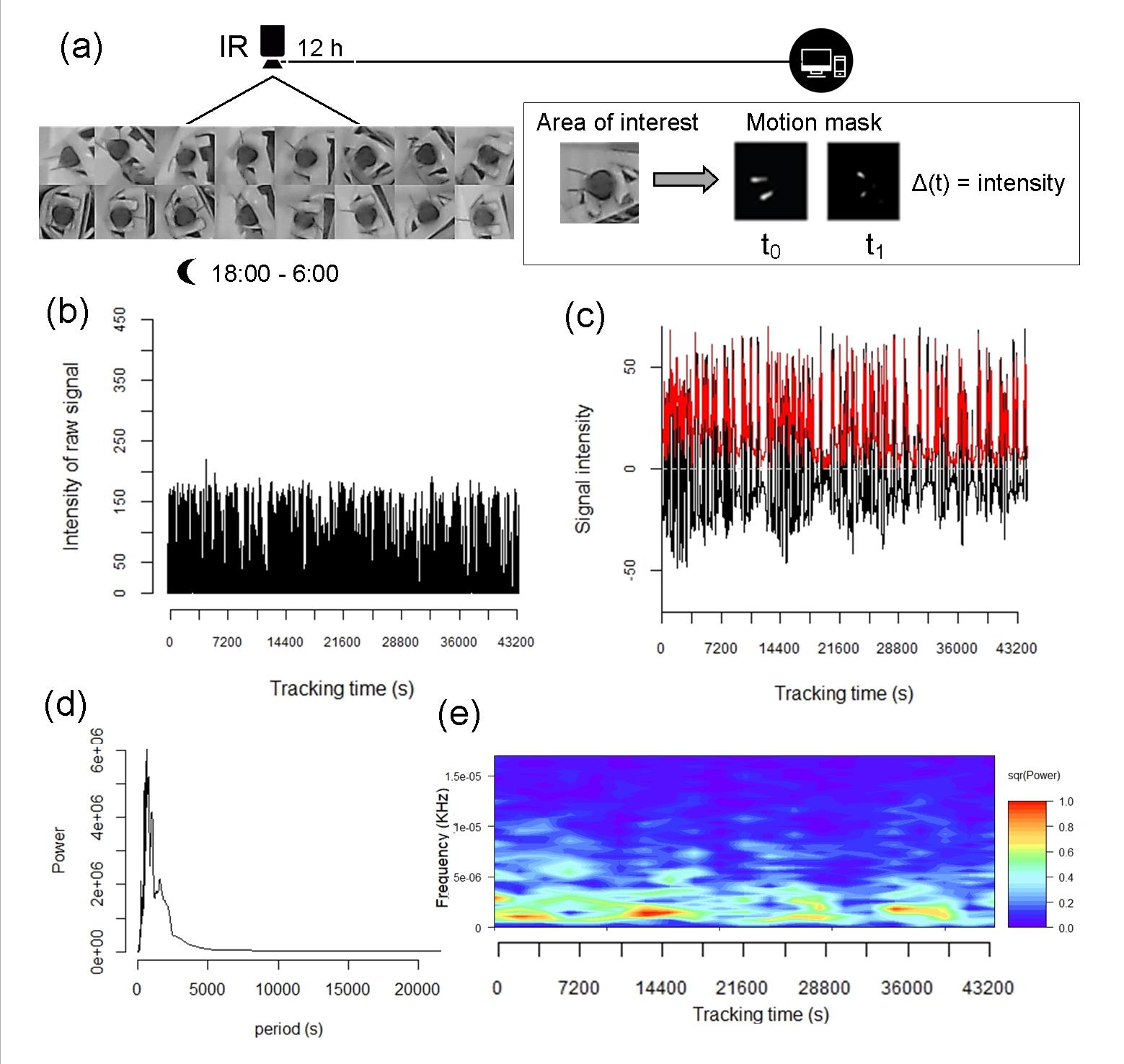


**Figure S1. The experimental device, signal processing and spectral analysis. (a)** Device capture of simultaneous recording of 16 bees during the scotophase (18:00-6:00) in darkness with IR light. The software tracks the antennal movement of each bee applying a motion mask that distinguishes the pixels (position and RGB value) of the image that change over time. An adaptive Gaussian mixture model for background subtraction was applied by the motion mask to separate the foreground (in white) from the background (in black) in the area of interest. The difference between foregrounds (a group of pixels) in two consecutive frames was calculated to quantify the magnitude of change and was defined as the intensity of the output raw signal. **(b)** Raw signal of a control bee showing the intensity of the antennal movement [on arbitrary units and tracking time showed in seconds (s)]. **(c)** Testing signal (black line) after the raw signal in panel *b* have been corrected with background signal and filtered for denoising. The red line is the envelope of the testing signal. **(d)** Periodogram of the testing signal in panel *c* after Fourier Transformation that allowed us to estimate the dominant cycle period and its associated power. **(e)** Spectrogram of the testing signal in panel *c* after short-term Fourier Transformation. The amplitude (√power) of the testing signal over time is displayed as a contour map drawn in the colour scale. Conventionally, red indicates the strongest signal component, while purple is the least strong.


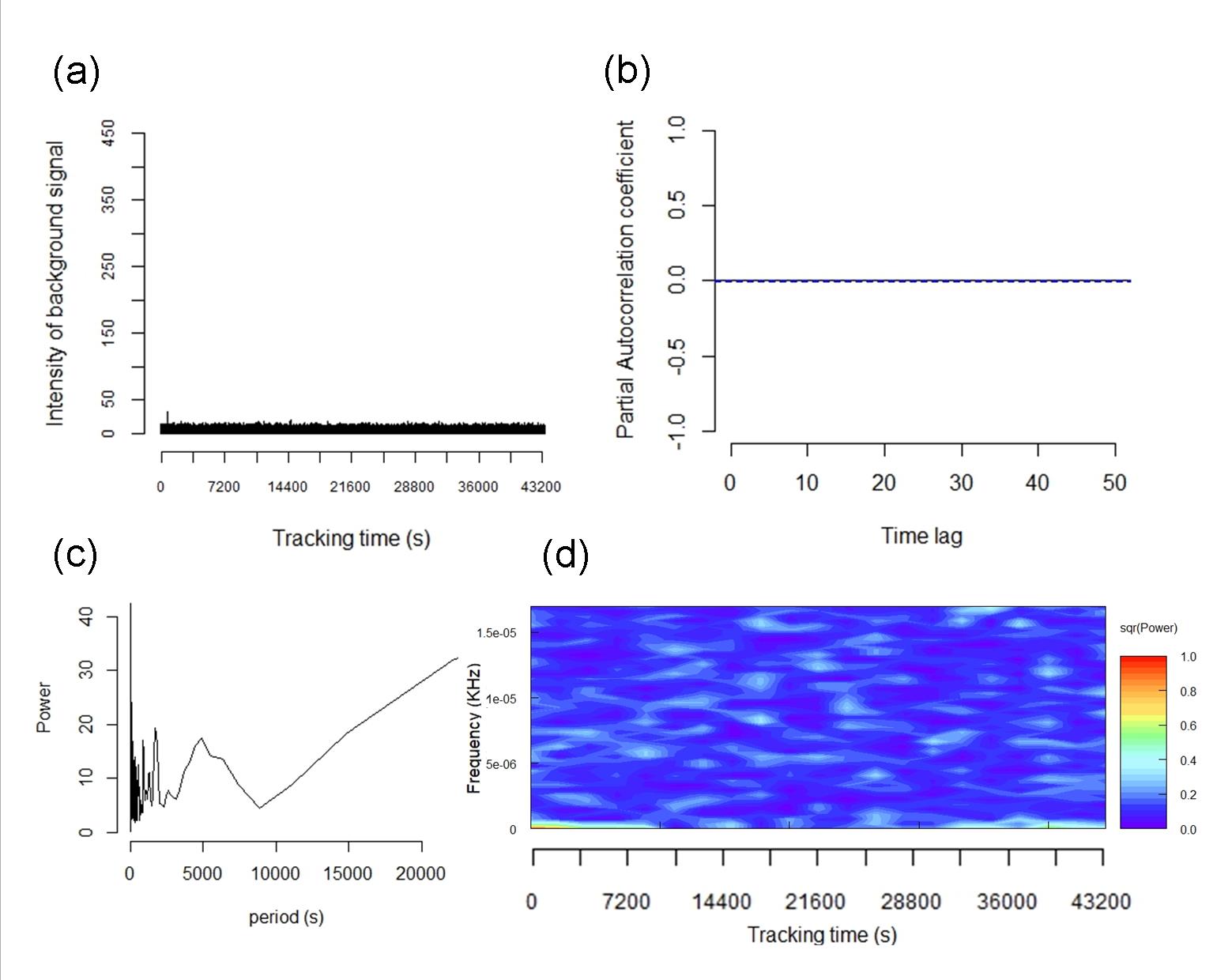


**Figure S2.** **Blank film and background signal.** To establish the background baseline of noise for testing signals (Fig S1), we used a blank recording. Tracking time is shown in seconds (s). **(a)** Tracking without bee (non-biological raw signal) during the scotophase of a bee in darkness with IR light. **(b)** Partial autocorrelation plot of the background signal of the panel *a*. Lag 0 is fixed at 1 by convention. The correlogram is plotted with their confidence interval (95%) with two dashed blue lines around zero in the coefficient axis. **(c)** Periodogram of the background signal of the panel a. **(d)** Spectrogram of the background signal of the panel a.


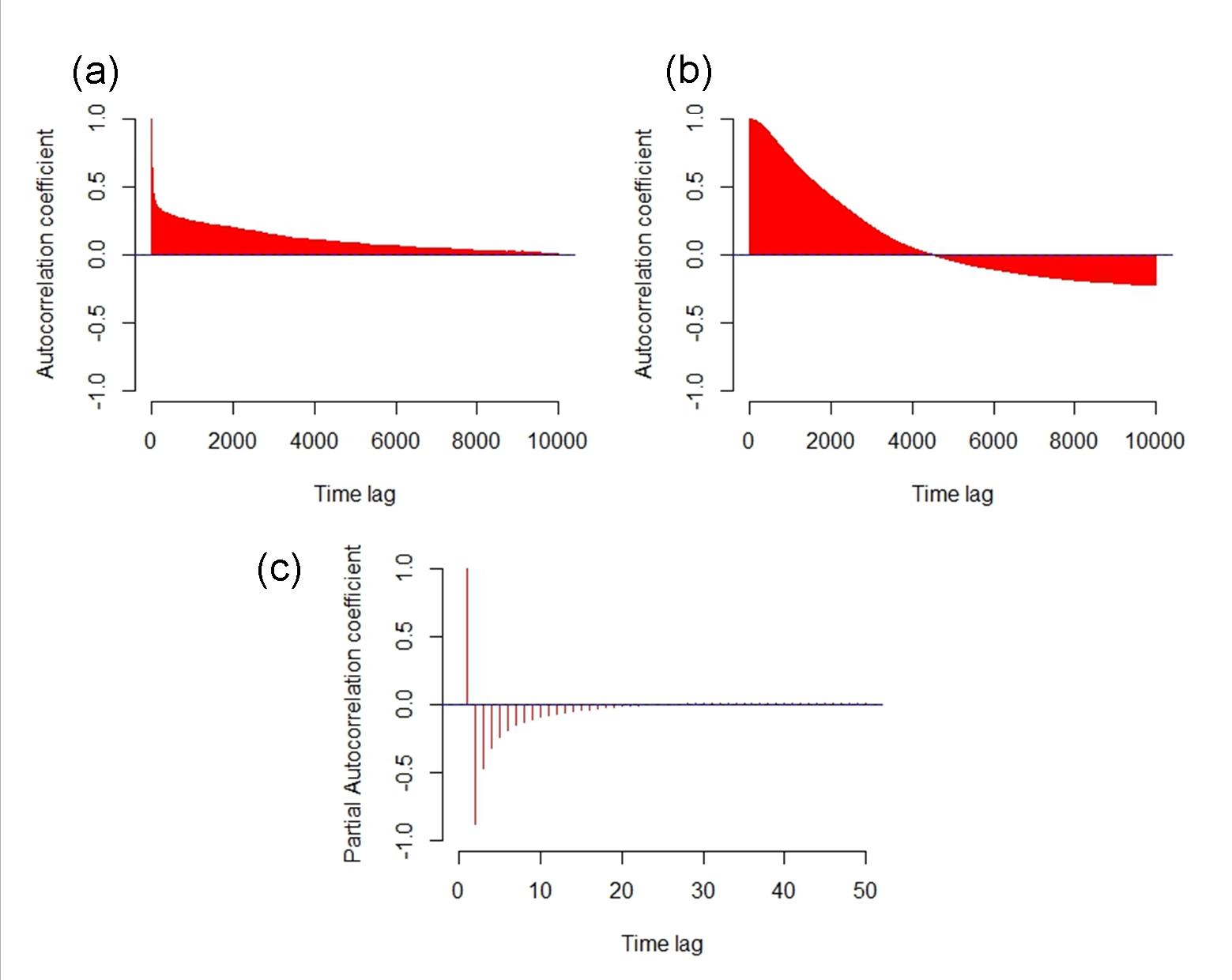


**Figure S3**. **Autocorrelation analysis to confirm periodicity of the biological signal.** Correlograms of a control bee. Lag (t+1) zero is fixed at 1 by convention. Each correlogram is plotted with its confidence interval (95%) with two dashed blue lines around zero in the coefficient axis. **(a)** Autocorrelation plot of the raw signal in panel *b* of Fig. S1. **(b)** Autocorrelation plot of the testing signal in panel *c* of Fig S1. **(c)** Partial Autocorrelation plot of the testing signal in panel *c* of Fig. S1.


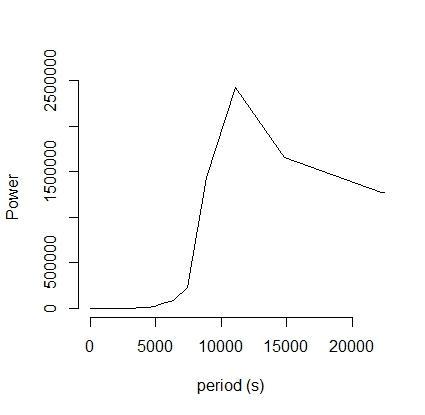


**Figure S4. Analysis of the amplitude modulation of the signal.** Periodogram generated from the envelope (red line) of the biological testing signal (black line) in panel *c* of Fig. S1. This spectral analysis (applying Fourier Transformation) of the filtered envelope allowed us to estimate the dominant cycle period of the modulation process and its associated power.


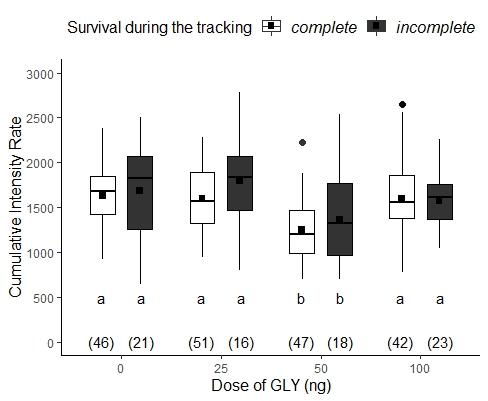


**Figure S5. Survival during tracking does not affect the intensity of antennal activity.** Dispersion of cumulative intensity rates of bees during scotophase displayed according to GLY exposure (acute doses of 0, 25, 50 and 100 ng) and their survival during the antennal tracking (boxplots white for complete survival and black for incomplete survival). The number of assessed bees per group is shown in brackets. Different letters indicate significant differences among doses of GLY (GLMM model: Cumulative Intensity Rate ~ [GLY] + Survival + (1|day), N = 264. Variance structure: < 0.01% among days. [GLY] term: F (3, 244) = 13.61, P < 0.001. Tukey test in Table S1. Survival term: F (1, 243) = 1.37, P = 0.244).


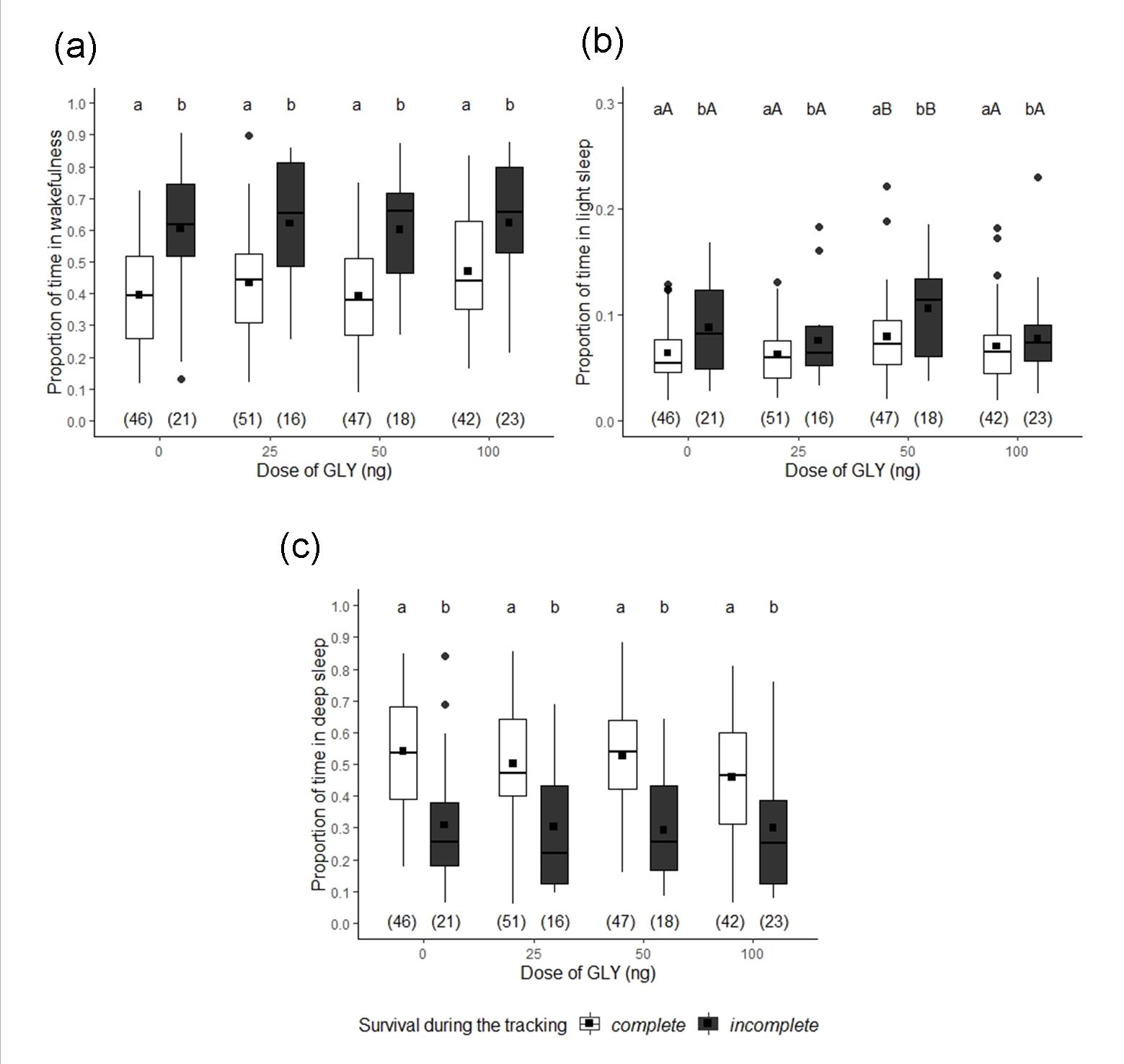


**Figure S6. Survival during tracking affects the proportion of time invested in rest.** Dispersion of proportions of time invested per forager bee in each stage of the resting-awakening cycle [**(a)**: wakefulness, **(b)**: light sleep and **(c)**: deep sleep] during scotophase displayed according to GLY exposure (acute doses of 0, 25, 50 and 100 ng) and their survival during the antennal tracking (boxplots white for complete survival and black for incomplete survival). The number of assessed bees per group is shown in brackets. Different lowercase letters indicate significant differences among survival status and different capital letters indicate significant differences among doses of GLY, but no capital letter indicates no significant differences. (GLMM models: prop. of time ~ [GLY] + Survival + (1|day), N = 264. Variance structure: < 0.01% among days. **(a)** [GLY] term: χ^2^ (3) = 3.44, P = 0.329. Survival term: χ^2^ (1) = 39.75, P < 0.001. **(b)** [GLY] term: χ^2^ (3) = 14.12, P = 0.003. Survival term: χ^2^ (1) = 15.37, P < 0.001. Tukey test in Table S2. **(c)** [GLY] term: F (3, 243) = 1.02, P = 0.384. Survival term: F (1, 246) = 64.63, P < 0.001).


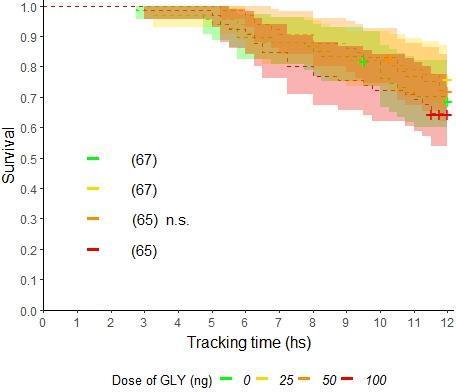


**Figure S7. Survival of honey bees during the record of its antennal movement.** The proportion of survival of bees during the recording period (12 h) from all 17 days of the experiment after acute exposure to GLY (0, 25, 50 and 100 ng). Bees were considered dead if they were more than 15 minutes quiescent and it was confirmed the morning after. Survival curves are plotted with their confidence interval (95%) for each dose. The number of assessed bees is shown in brackets. Fitting of data to CPH model [survival prop. ~ [GLY] + strata(day)]. The curves are plotted with different colours per dose: green for 0 ng (control) and a yellow-red gradient for increasing dose of GLY. The + indicates time points with censoring data. n.s. for no significant differences among groups.

**Table S1. Multiple *post hoc* pairwise comparisons for the proportion of time between pair of antennal movement states (wakefulness, light sleep and deep sleep).**

| z value / p-value* | wakefulness | light sleep | deep sleep |
| --- | --- | --- | --- |
| wakefulness |  | **< 0.001** | **0.036** |
| light sleep | 28.48 |  | **< 0.001** |
| deep sleep | -2.48 | 25.69 |  |

***** Tukey tests. p-value was corrected with Bonferroni procedure (significant differences in bold).

**Table S2. Multiple *post hoc* pairwise comparisons for the average dominant period between pair of treatments (dose of GLY).**

| z value (samples size) / p-value* | | | | |
| --- | --- | --- | --- | --- |
| Dose of GLY (ng) | 0 | 25 | 50 | 100 |
| 0 |  | 0.164 | 0.057* | 0.427 |
| 25 | -2.07 (134) |  | 0.963 | 0.947 |
| 50 | -2.52 (132) | -0.48 (134) |  | 0.737 |
| 100 | -1.52 (132) | 0.55 (132) | 1.02 (130) |  |

* Tukey tests. p-value was corrected with Bonferroni procedure (asterisk for marginal significant difference: P < 0.1).

**Table S3. Multiple *post hoc* pairwise comparisons for the average cumulative activity intensity between pair of treatments (dose of GLY).**

| z value (samples size) / p-value* | | | | |
| --- | --- | --- | --- | --- |
| Dose of GLY (ng) | 0 | 25 | 50 | 100 |
| 0 |  | 1.00 | **< 0.001** | 0.864 |
| 25 | -0.02 (134) |  | **< 0.001** | 0.870 |
| 50 | -5.49 (132) | -5.48 (134) |  | **< 0.001** |
| 100 | -0.78 (132) | -0.77 (132) | 4.68 (130) |  |

***** Tukey tests. p-value was corrected with Bonferroni procedure (significant differences in bold).

**Table S4. Multiple *post hoc* pairwise comparisons for the average signal-noise ratio 1 between pair of treatments (dose of GLY).**

| z value (samples size) / p-value* | | | | |
| --- | --- | --- | --- | --- |
| Dose of GLY (ng) | 0 | 25 | 50 | 100 |
| 0 |  | 0.662 | **< 0.001** | 0.146 |
| 25 | 1.13 (134) |  | **< 0.001** | 0.739 |
| 50 | 5.87 (132) | 5.06 (134) |  | **< 0.001** |
| 100 | 2.10 (132) | 1.01 (132) | -4.25 (130) |  |

***** Tukey tests. p-value was corrected with Bonferroni procedure (significant differences in bold).

**Table S5. Multiple *post hoc* pairwise comparisons for the average signal-noise ratio 2 between pair of treatments (dose of GLY).**

| z value (samples size) / p-value* | | | | |
| --- | --- | --- | --- | --- |
| Dose of GLY (ng) | 0 | 25 | 50 | 100 |
| 0 |  | 0.853 | **< 0.001** | **0.002** |
| 25 | 0.77 (134) |  | **< 0.001** | **0.009** |
| 50 | 5.23 (132) | 5.04 (134) |  | **0.002** |
| 100 | 3.57 (132) | 3.07 (132) | -3.57 (130) |  |

* Tukey tests. p-value was corrected with Bonferroni procedure (significant differences in bold).

**Table S6. Multiple *post hoc* pairwise comparisons for the average cumulative intensity rates between pair of treatments (dose of GLY).**

| z value (samples size) / p-value* | | | | |
| --- | --- | --- | --- | --- |
| Dose of GLY (ng) | 0 | 25 | 50 | 100 |
| 0 |  | 1.00 | **< 0.001** | 0.864 |
| 25 | -0.02 (134) |  | **< 0.001** | 0.870 |
| 50 | -5.49 (132) | -5.48 (134) |  | **< 0.001** |
| 100 | -0.78 (132) | -0.77 (132) | 4.68 (130) |  |

* Tukey tests. p-value was corrected with Bonferroni procedure (significant differences in bold).

**Table S7. Multiple *post hoc* pairwise comparisons for the proportion of time between pair of treatments (dose of GLY).**

| z value (samples size) / p-value* | | | | |
| --- | --- | --- | --- | --- |
| Dose of GLY (ng) | 0 | 25 | 50 | 100 |
| 0 |  | 0.812 | **0.036** | 0.999 |
| 25 | 0.89 (134) |  | **0.003** | 0.770 |
| 50 | -2.69 (132) | -3.52 (134) |  | **0.045** |
| 100 | -0.08 (132) | -0.96 (132) | 2.61 (130) |  |

* Tukey tests. p-value was corrected with Bonferroni procedure (significant differences in bold).

**SI References**

1. R Core Team, R: A language and environment for statistical computing. R Foundation for Statistical Computing, Vienna, Austria. URL: <https://www.R-project.org/>. (2017).
2. H. Wickham, ggplot2: Elegant Graphics for Data Analysis. Springer-Verlag New York. (2009).
3. Zwaka, H., Bartels, R., Gora, J., Franck, V., Culo, A., Götsch, M. & Menzel, R. Context odor presentation during sleep enhances memory in honeybees. *Curr. Biol.* 25(21), 2869-2874 (2015).
4. Vaidyanathan, P.P. Generalizations of the sampling theorem: Seven decades after Nyquist. IEEE Transactions on Circuits and Systems I: Fundamental Theory and Applications, 48(9), 1094-1109 (2001).
5. Jerri, A.J. The Shannon sampling theorem—Its various extensions and applications: A tutorial review. PIEEE, 65(11), 1565-1596 (1977).
6. Box, G.E.P., Jenkins, G.M. & Reinsel, G.C. Time Series Analysis, Forecasting and Control, 3rd ed. Prentice Hall, Englewood Clifs, NJ. (1994).
7. Brockwell, P.J. & Davis, R.A. Introduction to Time Series and Forecasting, 2nd. ed., Springer-Verlang. (2002).
8. Crawley, M.J. The R book. Imperial College London at Silwood Park. UK, 527-528. (2012).
9. Easdale, M.H. & Bruzzone, O. Anchored in average thinking in studies of arid rangeland dynamics–The need for a step forward from traditional measures of variability. *J. Arid Environ.* 116, 77-81 (2015).
10. Jenkins, D.G. & Watts, G. Spectral Analysis and Its Applications, Holden-Day. (1968).
11. Bloomfield, P. Fourier Analysis of Time Series, John Wiley and Sons. (1976).
12. Kleinfeld, D. & Mitra, P.P. Spectral methods for functional brain imaging. Cold Spring Harbor Protocols, 2014(3), pdb-top081075 (2014).
13. Stoddard, P.K. Application of filters in bioacoustics. In: Hopp, S. L., Owren, M. J. and Evans, C. S. (Eds), Animal acoustic communication. Springer, Berlin, Heidelberg,pp. 105-127 (1998).
14. Butterworth, S. On the theory of filter amplifiers. Wireless Engineer, 7(6), 536-541 (1930).
15. Sueur, J., Aubin, T. & Simonis, C. seewave: a free modular tool for sound analysis and synthesis. *Bioacoustics,* 18: 213-226 (2008).
16. Sejdić, E., Djurović, I. & Jiang, J. Time-frequency feature representation using energy concentration: An overview of recent advances. *Digit. Signal Process.* 19(1), 153-183 (2009).
17. Harris, F.J. On the use of windows for harmonic analysis with the discrete Fourier Transform. PIEEE, 66(1): 51-83 (1978).
18. Hall, M. Resolution and uncertainty in spectral decomposition. First Break, 24(12), 43-47 (2006).
19. Qin, Y., Qin, S. & Mao, Y. Research on iterated Hilbert transform and its application in mechanical fault diagnosis. *Mech. Syst. Signal Pr.* 22(8), 1967-1980 (2008).
20. Quinn, G.P. & Keough, K.J. Experimental design and data analysis for biologists. Cambridge University Press, London (2002).
21. Therneau, T.M. A Package for Survival Analysis in S. version 2.38, URL: <https://CRAN.R-project.org/package=survival>. (2015).
22. Therneau, T.M. & Grambsch,P.M. Modeling Survival Data Extending the Cox Model. Springer, New York. ISBN 0-387-98784-3 (2000).
23. Andersen, P. & Gill, R. Cox's regression model for counting processes, a large sample study*. Ann. Stat.* 10, 1100-1120 (1982).
24. Grambsch, P. & Therneau, T. Proportional hazards tests and diagnostics based on weighted residuals. *Biometrika*, 81, 515-26 (1994).
25. Tang, Y., Horikoshi, M. & Li, W. ggfortify: Unified Interface to Visualize Statistical Result of Popular R Packages. The R Journal 8.2:478-489 (2016).
26. Horikoshi, M. & Tang, Y. ggfortify: Data Visualization Tools for Statistical Analysis Results. <https://CRAN.R-project.org/package=ggfortify> (2016).
27. Pinheiro, J., Bates, D., DebRoy, S. &. Sarkar, D. R Core Team, nlme: Linear and Nonlinear Mixed Effects Models. R package version 3.1-131, URL: <https://CRAN.R-project.org/package=nlme>. (2017).
28. Zuur, A.F., Ieno, E.N., Walker, N.J., Saveliev, A.A. & Smith, G.M. Mixed effects models and extensions in ecology with R. Gail M., Krickeberg K., Samet J.M., Tsiatis A., Wong W., editors. New York, NY: Spring Science and Business Media. (2009).
29. Brooks, M.E., Kristensen, K., van Benthem, K.J.,Magnusson, A., Berg, C.W., Nielsen, A., Skaug, H.J., Maechler, M. & Bolker, B.M glmmTMB Balances Speed and Flexibility Among Packages for Zero-inflated Generalized Linear Mixed Modeling. The R Journal, 9(2), 378-400 (2017).
30. Bates, D., Maechler, M,, Bolker, B. & Walker, S. Fitting Linear Mixed-Effects Models Using lme4. J. Stat. Softw. 67(1), 1-48. doi:10.18637/jss.v067.i01. (2015).
31. Hothorn, T. & Bretz, F. Westfall, Simultaneous Inference in General Parametric Models. Biometrical J. 50(3), 346-363 (2008).
32. Zeileis, A. & Hothorn, T. Diagnostic Checking in Regression Relationships. R News 2(3), 7-10. URL <https://CRAN.R-project.org/doc/Rnews/> (2002).
33. Wright, S.P. Adjusted P-values for simultaneous inference. *Biometrics*, 48, 1005–1013. doi: 10.2307/2532694. (1992).
34. Shaffer, J.P. Multiple hypothesis testing. *Annu. Rev. Psychol*. 46, 561–584. doi: 10.1146/annurev.ps.46.020195.003021. (1995).
